# Efficacy and safety of polymyxin B in carbapenem-resistant gram-negative organisms infections

**DOI:** 10.1186/s12879-021-06719-y

**Published:** 2021-10-04

**Authors:** G. L. Xia, R. L. Jiang

**Affiliations:** 1 Department of Intensive Care Unit, The First Affiliated Hospital of Zhejiang Chinese Medical University (Zhejiang Provincial Hospital of Traditional Chinese Medicine), Hangzhou, China; 2 Department of Intensive Care Unit, The First Affiliated Hospital of Zhejiang Chinese Medical University (Zhejiang Provincial Hospital of Traditional Chinese Medicine), NO. 54 Youdian Road, Hangzhou, 310006 China

**Keywords:** Polymyxin B, Carbapenem-resistant gram-negative organisms, Infection, Rational drug use, Adverse reactions

## Abstract

**Objective:**

To investigate how to use polymyxin B rationally in order to produce the best efficacy and safety in patients with carbapenem-resistant gram-negative organisms (CRO) infection.

**Methods:**

The clinical characteristics and microbiological results of 181 patients caused by CRO infection treated with polymyxin B in the First Affiliated Hospital from July 2018 to May 2020 were retrospectively analyzed. The bacterial clearance rate, clinical efficacy, adverse drug reactions and 28 days mortality were evaluated.

**Results:**

The overall effective rate of 181 patients was 49.72%, the total bacterial clearance rate was 42.0%, and the 28 day all-cause mortality rate was 59.1%. The effective rate and bacterial clearance rate in the group of less than 24 h from the isolation of CRO to the use of polymyxin B were significantly higher than those in the group of more than 24 h. Logistics multivariate regression analysis showed that the predictive factors for effective treatment of CRO with polymyxin B were APACHEII score, duration of polymyxin B treatment, combination of polymyxin B and other antibiotics, and bacterial clearance. 17 cases (9.36%) of acute kidney injury were considered as polymyxin B nephrotoxicity and 4 cases (23.5%) recovered after polymyxin B withdrawal. After 14 days of polymyxin B use, 3 cases of polymyxin B resistance appeared, and there were 2 cases of polymyxin B resistance in the daily dose 1.5 mg/kg/day group.

**Conclusion:**

For CRO infection, the treatment of polymyxin B should be early, combined, optimal dose and duration of treatment, which can achieve better clinical efficacy and microbial reactions, and reduce the adverse reactions and drug resistance.

## Introduction

In recent years, with the wide application of broad-spectrum antibiotics in clinic and the poor control of nosocomial infections, the bacterial resistance rate has increased year by year, which has become a serious global problem. In particular, carbapenem-resistant gram negative organisms (CRO) have high resistance to conventional antibiotics, which increases the difficulty of treatment [1, 2]. Due to its unique chemical structure, polymyxin B can destroy the outer membrane integrity of Gram-negative bacilli [3]. The toxicity of polymyxin B may be alleviated due to the improvement of preparation quality, which promotes its clinical application [4]. Both domestic and foreign guidelines recommend polymyxin B as an important therapeutic drug for the infection of carbapenem-resistant bacterial strains such as carbapenem-resistant Enterobacteriaceae (CRE), carbapenem-resistant *Acinetobacter baumannii* (CRAB), and carbapenem-resistant *Pseudomonas aeruginosa* (CRPA) [5, 6].

Sepsis is defined as life-threatening organ dysfunction caused by the imbalance of immune response to infection, which is extremely dangerous, with a high mortality rate of 45–50% [7, 8]. According to epidemiology, the most common infection site of sepsis is the lung, followed by abdominal, gastrointestinal and blood flow infections. Most patients are infected at two or more sites simultaneously [9]. Gram-negative bacteria are the most common pathogens of sepsis, and *Klebsiella pneumoniae*, *Acinetobacter baumannii*, *Pseudomonas aeruginosa* and *Escherichia coli* are the main pathogens in China [4].

It has been reported that the all-cause mortality of carbapenem-resistant *Klebsiella pneumoniae* was 34.1–52.8% [10]. The data of 2019 CHINET China Bacterial Resistance Monitoring Network showed that the resistance rate of *Klebsiella pneumoniae* to carbapenems increased from 4.9% in 2013 to 10.9% in 2019. The resistance rates of *Acinetobacter baumannii* and *Pseudomonas aeruginosa* to carbapenems were as high as 56% and 19.1% [11]. The production of drug-resistant bacteria increases the pressure of antimicrobial drug selection, and sepsis caused by drug-resistant bacteria is a major problem in clinical treatment [12]. With the increase of fatal CRO infection, the ‘rigid demand’ for polymyxin B is increasing. However, the nonstandard use of polymyxin B by clinicians leads to the generation of mcr-1 gene resistant to polymyxin B [13]. We must consider how to standardize the rational use of polymyxin B, otherwise CRO infection may be drug-free. At present, there are few studies on how to regulate the rational use of polymyxin B to produce the optimal efficacy and safety. This study reviewed the clinical and microbiological data of 181 patients with sepsis caused by CRO infection treated with polymyxin B, and discussed how to standardize and rationally use polymyxin B to produce the optimal efficacy and safety for patients with CRO infection, so as to provide a basis for clinical application of polymyxin B.

## Methods

### Study subjects

A total of 181 CRO patients with sepsis who were treated with polymyxin B for injection (≥ 3 days) in the First Affiliated Hospital of Zhejiang Chinese Medical University from July 2018 to May 2020 were selected. The included patients were selected from all hospital wards, as long as they were treated with polymyxin B. This study was approved by the medical ethics committee of the hospital that waived the need for informed consent, due to its retrospective design. We confirm that all methods were carried out in accordance with relevant guidelines and regulations. Inclusion criteria: Patients (age ≥ 15 years) with all types of CRO infections according to etiological examination or clinical considerations, consistent with Sepsis-3 diagnostic criteria [7]. Exclusion criteria: Those who died within 3 days or 48 h after polymyxin B administration.

### Treatment plan

If the patient has carbapenem resistant bacterial infection or CRO infection is highly considered, or Gram-negative bacterial infection with ineffective drug treatment, we use polymyxin B to fight the infection. We selected the standard broth microdilution method recommended by EUCAST for the drug sensitivity test of polymyxin B and colistin. The European Commission for antimicrobial susceptibility testing suggested that polymyxa B adopt sensitivity (S ≤ 2 mg/L) and drug resistance (R > 2 mg/L) as the clinical break point. We also adopt this standard of the Minimum inhibitory concentration of antibiotics (MIC). Patients received polymyxin B (polymyxin sulfate B for injection, trade name: Yale, Shanghai first biochemical pharmaceutical Co., Ltd.) monotherapy or in combination with other antibiotics (such as carbapenems and Tigecycline) for at least 3 days, including loading dose (LD).

### Observed indexes

The data was extracted from the patient’s medical records and the hospital database, which included the information on the clinical characteristics, previous diseases, infection sites, bacterial culture and drug sensitivity test results, types and duration of antibiotics, complications, acute physiology and chronic health evaluation II (APACHE II) score before and after polymyxin B treatment [14], clinical outcome, biochemical indexes, microbial evaluation, curative effect evaluation, safety evaluation, 28 days mortality, etc. The day of withdrawal or death was the end point of the observation, and the follow-up end point was 28 days after the polymyxin B administration. The day of withdrawal: according to the clinical symptoms, inflammatory indexes and microbiological results of the patients, polymyxin B had poor effect after 72 h of use, resulting in the replacement of other antibiotics or good anti infection effect, but the course of treatment had reached the withdrawal indication.

### Definition

#### Efficacy evaluation criteria

(1) Cure: infection control, the inflammatory indicators returned to normal and no longer recurrence; (2) Improvement: patients’ inflammatory indexes decreased, body temperature decreased, and clinical manifestations such as decreased ventilator parameters and improved vital signs; (3) Invalid: no improvement was found in patients, including inflammatory indicators and clinical manifestations; Cure and improvement is effective.

#### Microbiological evaluation definition

(1) Clearance: Clearance refers to the infection site after administration of bacterial culture did not find the original pathogenic bacteria. (2) Uncleared: the amount of bacteria is not reduced or reduced but not completely cleared. The primary outcome was clinical efficacy, and the secondary outcome was microbial clearance.

#### Safety assessment

Adverse reaction related to polymyxin B was closely observed, including skin pigmentation, acute renal injury [15] and neurotoxicity, as well as contact dermatitis, pruritus, drug fever and other reactions.

### Statistical analysis

SPSS 23.0 software (SPSS Inc., USA) was used for statistical analyses. The variables distribution was assessed by the Kolmogorov–Smirnov test. The data with a non-normal distribution were assessed with Mann–Whitney test and the median and selected centiles (25th to 75th) value was given. The data with a normal distribution were assessed with the Student t-test. The Chi-square test or Fisher exact test was used to analyze the categorical variables, which were presented as proportions. Univariate analysis of statistically significant factors and important clinical factors were included in the Logistic regression model. Multivariate logistic regression was used to analyze clinically effective independent risk factors, and Hosmer Lemeshow test was used to determine the goodness of fit of logistic regression model. A P-value < 0.05 was considered significant.

## Results

### Clinical features

15 cases of exfoliation were treated with polymyxin B for less than 3 days. So a total of 181 patients with CRO infections sepsis were retrospectively analyzed. There were 111 cases in hematology department, 58 cases in ICU, 12 cases in respiratory department, infection department and gastrointestinal department. 181 patients were aged from 15 to 93 years old, with an average of (61.1 ± 18.8) years old, including 135 males (76.24%); some patients had severe complications before polymyxin B treatment, including septic shock in 42 cases (23.2%), multiple organ dysfunctions in 40 cases (22.1%), and acute respiratory distress syndrome (ARDS) in 25 cases (13.8%) (see Table 1).

**Table 1:** Clinical characteristics of 181 patients with CRO infections

Characteristic	Findings
Age (year), mean ± SD	0.39 ± 0.17
Male, n (%)	135 (76.2)
APACHE II score, mean ± SD	19.25 ± 6.11
Distribution of departments, n (%)	
Hematology department	111 (61.3)
ICU	58 (32.0)
Other departments	12 (6.6)
Comorbidities, n (%)	
Chronic heart failure	52 (28.7)
Diabetes mellitus	45 (24.9)
COPD	34 (18.8)
Chronic kidney disease	31 (17.7)
Immunosuppressive status	25 (13.8)
Complications, n (%)	
Shock	42 (23.2)
MODS	40 (22.1)
ARDS	27 (14.9)
Infection types, n (%)	
Pneumonia	104 (57.5)
Bloodstream infection	36 (19.9)
Intestinal infection	35 (19.3)
Abdominal infection	9 (5.0)
Other infections	40 (22.1)
Multiple site infections	64 (35.4)
Responsible pathogens, n (%)	
K.P	91 (50.3)
P.A	58 (32.0)
* E. coli*	16 (8.8)
A.B	12 (6.6)
Multiple bacterial infections	50 (27.6)
Polymyxin B dose, mean ± SD	1.91 ± 0.33
Initial treatment, n (%)	70 (38.7)
Duration of polymyxin B treatment, days, median (IQR)	11 (6, 19)
Antibiotic combination, n (%)	167 (92.3)
Microbiological eradication, n (%)	76 (42.0)

APACHE II: acute physiology and chronic health evaluation II; ICU: intensive care unit; MODS: multiple organ dysfunction syndrome; ARDS: acute respiratory distress syndrome; COPD: chronic obstructive pulmonary disease; K.P.: *Klebsiella pneumonia*; P.A.: *Pseudomonas aeruginosa*; *E. coli*: *Escherichia coli*; E.fm: *Enterococcus faecium*; A.B.: *Acinetobacter baumannii*

### Type of infection and microbiological analysis

Infection site: 104 cases of lung, 36 cases of blood flow, 35 cases of intestinal tract, 9 cases of abdominal cavity, 3 cases of urinary tract, 2 cases of pleural effusion, 1 case of scrotal abscess, 1 case of parotid abscess, 33 cases of unknown infection site. The effective rate of polymyxin B in the treatment of Pneumonia was slightly higher than that of Bloodstream infection, and the clearance rate was slightly lower than that of Bloodstream infection. The mortality of the two groups was similar, and the difference was not statistically significant (P > 0.05) (see Table 2).

**Table 2:** Comparison of effective rate and clearance rate in different groups

Group	n	Effective rate (n (%))	P-value	Clearance rate (n (%))	P-value
Infection types					
Pneumonia	104 (44.2)	46 (44.2%)	0.395	35 (33.7%)	0.387
Bloodstream infection	36 (44.2)	13 (36.1%)		15 (41.7%)	
Prognosis					
Survival	74 (44.2)	71 (95.9%)	≤ 0.001	56 (75.7%)	≤ 0.001
Death	107 (44.2)	19 (17.8%)		20 (18.7%)	
Time from carbapenem resistance to PB use (h)					
< 24	55 (44.2)	33 (60.0%)	0.008	30 (54.5%)	0.016
> 24	93 (44.2)	35 (37.6%)		32 (34.4%)	

### Clinical efficacy and prognosis evaluation

181 patients were treated with polymyxin B, 90 cases were effective, the effective rate was 49.72%; 76 cases were cleared; the total clearance rate was 42.0%. 107 patients died within 28 days (including automatic discharge). The 28 day all-cause mortality was 59.1%. The clinical effective rate and bacterial clearance rate of the death group were significantly lower than those of the survival group (P < 0.05), as shown in Table 2.

### Efficacy evaluation according to the timing of polymyxin B treatment

The clinical effective rate and bacterial clearance rate in the group of less than 24 h from the isolation of carbapenem resistant bacteria to polymyxin B use were significantly higher than those in the group of more than 24 h (see Table 2).

### Efficacy evaluation according to the duration of polymyxin B treatment

The average duration of polymyxin B in 181 patients was 11.27 ± 7.60 days, the effective group for 14.6 ± 8.18 days, and the ineffective group for 7.98 ± 5.23 days; compared with the 3–7 days group, the effective rate and bacterial clearance rate of the 8–14 days group and the > 14 day group were significantly higher (all P < 0.05). There was no significant difference in the effective rate and bacterial clearance rate between the treatment group of > 14 days and the treatment group of 8–14 days (P > 0.05) (see Table 3).

**Table 3:** Effect of polymyxin B in different duration, dose and medication mod on curative effect

Group	n	Effective rate (n (%))	Clearance rate (n (%))
Duration of treatment ( days)			
3–7	71	16 (16.8%)	12 (16.9%)
8–14	57	35 (61.4%)^a^	32 (56.1%)^a^
> 14	53	39 (73.5%)^a^	32 (60.4%)^a^
Daily dose (mg/kg/day)			
1.5	70	19 (27.1%)	25 (35.7%)
2.0	97	46 (47.4%)^b^	53 (54.6%)^b^
2.5	14	10 (71.4%)^b^	11 (78.6%)^b^
Medication mode			
Polymyxin B monotherapy	14	3 (28.57%)	2 (14.29%)
Polymyxin B combination	167	87 (52.1%)^c^	74 (44.3%)^c^
Carbapenems	39	20 (51.3%)	15 (38.5%)^d^
Tigecycline	61	15 (48.4%)	12 (38.7%)^d^
Fosfomycin	18	10 (55.6%)	9 (50.0%)^d^
Tigecycline + fosfomycin	30	19 (63.3%)	20 (66.7%)
Carbapenems + tigecycline	17	10 (58.8%)	8 (52.9%)^d^
Other	32	13 (41.9%)	10 (31.3%)^d^

In the group of treatment duration, compared with 3–7 days group, ^a^P < 0.05

In the group of daily dose, compared with 1.5 mg/kg/days group, ^b^P < 0.05

In the group of medication mode, compared with polymyxin B monotherapy group, ^c^P < 0.05; compared with polymyxin + tigecycline + fosfomycin group, ^d^P < 0.05

### Efficacy evaluation according to polymyxin B daily dosage

Compared with the daily dose of polymyxin B 1.5 mg/kg/day group, the effective rate and bacterial clearance rate of 2.0 mg/kg/day group and 2.5 mg/kg/day group were significantly increased (all P < 0.05). The effective rate and clearance rate of 2.5 mg/kg/day group were higher than that of 2.0 mg/kg/day group, but the difference was not significant (P > 0.05) (see Table 3).

### Efficacy evaluation according to polymyxin B combined regimen

The effective rate and clearance rate of polymyxin B combined with other antibiotics were higher than that of polymyxin B monotherapy, the difference was significant (P < 0.05); the clearance rate of polymyxin B combined with tigecycline and fosfomycin was the highest, reaching 66.7%, which was significantly higher than other Polymyxin B combination (P < 0.05). The effective rate of polymyxin B + tigecycline + fosfomycin group was 63.3%, but there was no significant difference with other groups (P > 0.05) (see Table 3).

### Predictive factors of the clinical efficacy

Univariate regression analysis showed that the predictive factors for the efficacy of polymyxin B in the treatment of G-bacteria were APACHEII score, the dose of polymyxin B, initial treatment, the duration of treatment of polymyxin B, the combination of polymyxin B with other antibiotics, and bacterial clearance. The above included multivariate regression analysis showed that the predictive factors for the efficacy of polymyxin B in the treatment of G-bacteria were APACHEII score, the duration of treatment of polymyxin B, the combination of polymyxin B with other antibiotics, and bacterial clearance (see Table 4).

**Table 4:** Univariate and multivariate analysis of factors associated with clinical cure in 181 patients with CRO infections

Characteristic	Univariate analysis		Multivariate analysis	
	Odds ratio (95% confidence interval)	P-value	Odds ratio (95% confidence interval)	P-value
Age (years)	1.006 (0.987, 1.026)	0.547		
Male	1.111 (0.563, 2.193)	0.761		
APACHE II score	0.944 (0.897, 0.993)	0.027	0.880 (0.811 0.955)	0.002
Comorbidities				
Chronic heart failure	0.587 (0.322, 1.067)	0.081		
Diabetes mellitus	0.975 (0.542, 1.755)	0.933		
COPD	0.761 (0.390, 1.483)	0.422		
Chronic kidney disease	1.069 (0.574, 1.990)	0.833		
Immunosuppressive status	1.017 (0.550, 1.879)	0.957		
Infection types				
Pneumonia	0.595 (0.328, 1.078)	0.087		
Bloodstream infection	0.499 (0.235, 1.061)	0.071		
Multiple site infections	0.530 (0.248, 1.131)	0.101		
Responsible pathogens				
K.P	0.893 (0.498, 1.603)	0.705		
P.A	0.508 (0.258, 1.000)	0.050		
*E. coli*	2.395 (0.797, 7.197)	0.120		
A.B	0.236 (0.049, 1.143)	0.073		
Multiple bacterial infections	0.393 (0.144, 1.073)	0.068		
Polymyxin B dose	2.367 (1.403, 3.991)	0.001	1.376 (0.558, 3.394)	0.488
Initial treatment	2.169 (1.177, 3.995)	0.013	1.643 (0.662, 4.077)	0.284
Duration of polymyxin B treatment(days)	1.168 (1.103, 1.236)	≤ 0.001	1.157 (1.077, 1.243)	≤ 0.001
Antibiotic combination	3.987 (1.074, 14.811)	0.039	3.853 (0.627, 23.668)	0.145
Microbiological eradication	26.541 (11.510, 61.202)	≤ 0.001	21.345 (8.007, 56.901)	≤ 0.001

A.B.: *Acinetobacter baumannii*; APACHE II: acute physiology and chronic health evaluation II; COPD: chronic obstructive pulmonary disease; *E. coli*: *Escherichia coli*; E.fm: *Enterococcus faecium*; K.P.: *Klebsiella pneumonia*; P.A.: *Pseudomonas aeruginosa*

### Safety assessment

30 patients (16.57%) appeared skin pigmentation during medication, 13 patients in the treatment > 14 days group and 12 cases in the daily dose of 2.5 mg/kg/day group, mainly in the head, neck and limbs, pigmentation gradually subsided and returned to normal after polymyxin B withdrawal. Neurotoxic reaction in 3 cases (1.66%) manifesting as for finger and toe numbness, and after drug withdrawal finger sensory abnormalities disappeared. 17 cases (9.36%) of acute kidney injury were considered as polymyxin B nephrotoxicity when the infection was controlled but the renal function was still damaged. Renal function recovered in 4 cases (23.5%) after polymyxin B withdrawal. And no drug-induced allergic reaction, liver, blood and other adverse reactions were found during treatment with polymyxin B. Statistics of all patients with bacteriological susceptibility results, there were 3 cases polymyxin B resistance after the polymyxin B treatment duration > 14 days and 2 cases in the daily dose of 1.5 mg/kg/day group. And thus ceftazidime/avermectin were used instead to anti-infection. Other groups did not appear polymyxin B resistance.

## Discussion

In this study, Pneumonia was still the main infection, accounting for 57.5%, followed by Bloodstream infection. A total of 227 strains of pathogenic bacteria were detected. *Klebsiella pneumoniae* was the most isolated strain, followed by *Pseudomonas aeruginosa*, *Escherichia coli* and *Acinetobacter baumannii*, and the other two or three strains were infected simultaneously. Polymyxin B has the highest sensitivity, and the combination regimen based on polymyxin B is the preferred treatment for systemic or local infections caused by the above three bacteria [7]. Since the cationic cyclic peptide in the structure of polymyxin B can bind to the sulfate ion in the active center of endotoxin, resulting in the inactivation of endotoxin, which has the effect of antagonism against endotoxin. So polymyxin B has important value in the clinical treatment of endotoxin-induced sepsis [16, 17].

The clinical data of 181 patients with sepsis treated with polymyxin B were retrospectively analyzed in our study. The clinical manifestations, laboratory tests and imaging findings of 90 patients were significantly improved. The effective rate was 49.7%, and the bacterial clearance rate was 42.0% (76/181). By evaluating the effect of using time and efficacy of polymyxin B, it was found that the clinical effective rate and bacterial clearance rate were significantly higher than those of > 24 h when the time from separation of carbapenem-resistant bacteria to use polymyxin B was less than 24 h, indicating that the earlier the target anti-infection of polymyxin B is, the better the clinical effect is. It is necessary to strengthen the close connection between microbial laboratories and clinical departments, and to treat anti-infection according to microbiological results as soon as possible. The new prediction indexes and rapid diagnostic techniques can shorten the time for determining the use of polymyxin B, benefit those patients who really need to use polymyxin B as soon as possible, and limit those patients who do not have indications to be treated with polymyxin B [18–21]. The guidelines for sepsis also show [11] that once the diagnosis of sepsis and etiological examination are made clear in clinic, anti-infection treatment should be carried out as soon as possible, and the antibiotic regimen should be adjusted early according to the results of bacterial susceptibility. If delayed treatment, even if the drug selection is appropriate, it can still lead to increased mortality and prolonged hospitalization. For the duration of polymyxin B treatment, compared with the 3–7 days treatment group, the effective rate and bacterial clearance rate of the 8–14 days treatment group and the > 14 days treatment group were significantly increased (P < 0.05), but there was no significant difference between the latter two groups (P > 0.05), but the adverse reactions of the > 14 days treatment group were more, and 3 patients were resistant to polymyxin B, indicating that the duration of polymyxin B is sufficient, but the longer the duration of treatment is not the better, the longer the duration of treatment is, the more likely to induce bacterial resistance to polymyxin B, and produce more adverse reactions.

Polymyxin B is a concentration-dependent antibiotic. According to its PK/PD target value, the consensus of the international guidelines for polymyxin B in 2019 [5] recommends that the steady-state AUCs of polymyxin B reach 50–100 (mg h)/L at 24 h, which is equivalent to the steady-state average blood concentration of Css, and reach 2–4 mg/L at 24 h. In the daily dose comparison of polymyxin B, the effective rate and bacterial clearance rate of daily dose 2 mg/kg/day group and 2.5 mg/kg/day group were significantly higher than those of daily dose 1.5 mg/kg/day group (P < 0.05), and there was no obvious difference between the first two groups (P > 0.05). However, the adverse reactions of daily dose 2.5 mg/kg/day group increased, and the daily dose 1.5 mg/kg/day group led to 2 patients with polymyxin B resistance, indicating that the dose of polymyxin B is sufficient, but the dose is not the greater the better. It needs to be calculated according to the patient’s weight. Low dose can induce bacterial resistance to polymyxin B, resulting in poor therapeutic effect. High dose is prone to produce more adverse reactions, such as skin pigmentation. Polymyxin B is mainly eliminated through non-renal pathway, so it doses not need to adjust the dose according to renal function. However, in patients with cystic fibrosis, PPK model found that the clearance of polymyxin B was related to CrCl, and patients with renal dysfunction need to increase the dose of polymyxin B [22]. Studies have shown that polymyxin B dose ≥ 200 mg/day can reduce 30 days mortality in patients with multidrug-resistant bacteria infection receiving renal replacement therapy [23]. But in general the daily total dose of polymyxin B should not exceed 200 mg/day to reduce adverse reactions and improve patient compliance [5], and our study also confirmed it. Studies have shown that the factors of treatment failure in severe patients with multidrug-resistant *Acinetobacter baumannii* (MDR-AB) bacteremia or pneumonia are small dose of polymyxin B (15,000 U/(kg day)), short duration of treatment, and not combined with sulbactam, which is consistent with the results of this study [24].

Considering the heterogeneous resistance of polymyxin B, it should be combined with other antibiotics to resist infection [25]. Clinical studies have suggested that in patients with KPC-producing *Klebsiella pneumoniae* bloodstream infection, polymyxin B combined with amikacin [26] can reduce mortality compared with polymyxin B monotherapy. This study also demonstrated that the effective rate and clearance rate of polymyxin B combined with other antibiotics were higher than those of polymyxin B monotherapy (P < 0.05). The combination regimen of polymyxin B + tigecycline + fosfomycin has the highest clearance rate and effective rate. There are many combinations of polymyxin B in clinical practice. If the clinical effect of a combination regimen is not good, it can be adjusted to polymyxin B combined with tigecycline and fosfomycin.

APACHE II score is an independent risk factor affecting the mortality of sepsis. The high score indicates that the more severe the patient is and the higher the mortality is [12]. Logistics multivariate regression analysis demonstrated that the effective predictive factors of polymyxin B in the treatment of CRO were APACHEII score, the duration of treatment of polymyxin B, the combination of polymyxin B and other antibiotics, and bacterial clearance. It showed that polymyxin B combinated with other antibiotics based on the appropriate duration of treatment could achieve the maximum bacterial clearance rate, reduce the APACHEII score and further decrease the mortality. After the withdrawal of polymyxin B, the skin pigmentation and neurotoxicity gradually subsided and returned to normal. Polymyxin B has low nephrotoxicity (9.36%) in this study and 23.6% acute kidney injury recovered after polymyxin B withdrawal, indicating that reasonable application of polymyxin B could achieve better clinical efficacy and microbiological reactions, and had less adverse reactions. However, unreasonable use would increase the resistance of bacteria and the occurrence of adverse reactions. According to the data of China Drug Resistance Surveillance Network in 2019, the resistance rates of *Acinetobacter baumannii* and *Pseudomonas aeruginosa* to polymyxin B were 2% and 1.3%, respectively, which were higher than those in 2018 [4]. With the excessive use of polymyxin B, the KPC-producing *Klebsiella pneumoniae* resistant to polymyxin B has been reported worldwide [27]. *Escherichia coli* also has resistance to polymyxin B [25], and there are Ceftazidime avibatan resistant bacteria [28]. The new antibacterial drugs have not been widely used in clinic, which should be paid attention to and require us to use polymyxin B rationally.

This study has some limitations. First, it is a single center retrospective analysis. Second, we did not monitor the plasma and tissue concentrations of polymyxin B and the lack of different doses of PK/PD data analysis. In order to better evaluate the efficacy of drugs, it is necessary to monitor the blood concentration of polymyxin B, and design prospective, multicenter, randomized controlled studies.

In conclusion, the rational use of polymyxin B is very important. Early, combined and optimal dosage and duration of treatment can achieve better clinical efficacy and microbial reactions, reduce adverse reactions and decrease the resistance to polymyxin B. We should also do a good job in the prevention and control of nosocomial infection, prevent the spread of drug-resistant bacteria between hospitals and regions, and cherish polymyxin B as a “life-saving drug” to avoid no drug available.

## Data Availability

The raw dataset is not available publically due to China privacy regulations. Applicants for any data must be prepared to conform to China privacy regulations.

## Abbreviations

CRO: Carbapenem-resistant gram-negative organisms
CRE: Carbapenem-resistant Enterobacteriaceae
CRAB: Carbapenem-resistant *Acinetobacter baumannii*
CRPA: Carbapenem-resistant *Pseudomonas aeruginosa*
EUCAST: The European Commission for antimicrobial susceptibility testing
MIC: The Minimum inhibitory concentration of antibiotics
APACHE II: Acute physiology and chronic health evaluation II
ICU: Intensive care unit
MODS: Multiple organ dysfunction syndrome
ARDS: Acute respiratory distress syndrome
COPD: Chronic obstructive pulmonary disease
K.P.: *Klebsiella pneumonia*
P.A.: *Pseudomonas aeruginosa*
*E. coli*: *Escherichia coli*
E.fm: *Enterococcus faecium*
A.B.: *Acinetobacter baumannii*
MDR-AB: Multidrug-resistant *Acinetobacter baumannii*
KPC: Carbapenemase produced in *Klebsiella pneumoniae*
PK/PD: Pharmacokinetic/pharmacodynamics
CrCl: Creatinine clearance rate
Css: Mean steady state plasma concentration
